# Is the calorie concept a real solution to the obesity epidemic?

**DOI:** 10.1080/16549716.2017.1289650

**Published:** 2017-05-09

**Authors:** Salvador Camacho, Andreas Ruppel

**Affiliations:** ^a^ Institute of Public Health, Universitätsklinikum Heidelberg, Heidelberg, Germany; ^b^ Alfred-Weber Institute, Universität Heidelberg, Heidelberg, Germany

**Keywords:** Obesity, calorie imbalance, obesity cause, hormonal imbalance, insulin, public health

## Abstract

**Background:** The obesity epidemic has been growing steadily across the whole world, and so far not a single country has been able to reverse it. The cause of obesity is stated by the World Health Organization as an energy imbalance between calories consumed and calories expended. However, growing evidence suggests that the calorie imbalance concept may not be sufficient to manage and reverse the obesity epidemic.

**Objective:** To discuss the use of the calorie imbalance concept and its elements as a tool for weight management as well as its possible negative consequences and implications for public health, with the aim to point toward the need of an updated concept for causes of obesity. This update should guide public health interventions more efficiently to limit obesity by preventing weight gain or promoting weight loss.

**Methods:** This is a literature reviews based on a semi-structured approach to determine the material to be examined.

**Results:** After revisiting general facts about fat generation and accumulation, we propose an updated concept for the causes of obesity including diet composition and hormonal regulation of fat metabolism.

**Conclusions:** We discuss how this updated concept could benefit the overall efficiency of strategies against obesity, and hypothesize how potential resistance to adopting this new view could be lowered.

## Background

The World Health Organization (WHO) defines obesity as an ‘abnormal or excessive fat accumulation that may impair health’, and states that ‘the fundamental cause of obesity and overweight is an energy imbalance between calories consumed and calories expended’ (n.b. Food energy is commonly expressed as ‘kilocalories [kcal]’ [1]. However, the common use is to name them just ‘calories [cal]’. In this article kcal and cal will be used interchangeably). This concept is deeply rooted in public opinion as the core reason for overweight and obesity. Most public health strategies aiming to tackle obesity are based on this concept, i.e. they aim to decrease caloric consumption, to increase calories expended or ideally a combination of both. However, the concept of energy balance or imbalance may be incomplete (n.b. Because the relevant energy unit is calories, energy balance, calorie balance, calorie imbalance and energy imbalance will be used interchangeably). It may even be argued that this concept could be one of the reasons why public health strategies have been so unsuccessful in reversing the obesity epidemic [2]. The energy balance will be referred to as ‘concept 1’ in this article.

An alternative, although not exclusive, conceptual frame links obesity to the insulin-dependent regulation of fat generation and takes note of the different metabolic pathways to degrade macronutrients, i.e. carbohydrates (CHO), fat and proteins. This article revisits causes for obesity in the light of the latter frame and its concepts, e.g. glycemic index, and will discuss how this frame can overcome some of the weaknesses of concept 1, with the aim to more efficiently guide public health interventions to limit obesity by either preventing weight gain or promoting weight loss. This can be seen as a hormonal-balance concept, and it will here be referred to as concept 2. Strengths and weaknesses of both concepts will be discussed.

## Methods

This is a narrative literature review covering evidence about obesity, its causes, control efforts and public health strategies. There are several approaches to study the causes of obesity, and this diversity provokes limitations. Therefore, this review does not claim to analyze the literature comprehensively, nor can we fully exclude potential risks of drawing conclusions on a possibly less-than-perfect basis. However, the chosen approach appears adequate to integrate complex interactions that apparently determine obesity [3].

## Calorie imbalance as the suggested cause for obesity

Concept 1 has its roots in two laws of thermodynamics, i.e. the first law known as the ‘conservation law’ and the second law also known as the ‘dissipation law’. The first law states that in a closed system in thermal equilibrium, the form of energy may change but the total is always conserved [4]. This has been interpreted as a caloric balance, i.e. ‘calories in should equal calories out’. Therefore, if you gain weight you are either eating too much or moving too little, and in order to lose weight you would have to ‘eat less and/or move more’. Despite its apparently straightforward logic, this concept is not complete and the second law also needs to be considered. This states that ‘in any irreversible process, the entropy must increase and balance is not expected’ [4]. That means that for digestive processes where food turns into bolus, but bolus cannot be converted back into food, some of the input energy is irreversibly lost during the metabolic process and results in thermogenesis.

## Concept 1 to avoid or reduce overweight and obesity

Because concept 1 assumes that a positive energy balance results in fat mass, individuals aiming to prevent weight gain should avoid a positive balance. Of the same tenor, individuals aiming to lose weight should look for a negative balance. Dietary guidelines commonly suggest the calories deficit needs to be in the range of 500–750 kcal per day for an adult to lose weight [5]. This value is based on the ‘3500 kcal rule’, also known as the ‘Wishnofsky rule’ [6], which is still used as the basis for some guidelines, publications and nutrition textbooks [7], despite its inaccuracy and very limited effectiveness [8]. The recommended deficit tacitly assumes ‘that a calorie is a calorie’ independently of its source [4], hence ignoring the second rule of thermodynamics. When the different values of catabolism-induced thermogenesis are considered for each macronutrient [9], ‘a calorie is a calorie’ may no longer hold true, but probably many among the common population and even some nutrition professionals ignore this [7,10]. By consequence, inaccurate assessments and recommendations for weight management may result.

Thus, concept 1 may lead to the misperception that total calorie intake is more important than the source of the calories and nutrient balance. Therefore, an individual trying to lose weight may become vulnerable to malnutrition when focusing only on calories. For example, restricting energy intake to 2000 kcal per day [5], if the resulting diet consists only of industrialized food (n.b. Products made from processed substances, extracted or refined from whole foods… [They are] very durable, palatable, and ready to consume… [They] are typically energy-dense, have a high glycemic load, are low in dietary fibers, micronutrients, and phytochemicals, and are high in unhealthy types of dietary fat, free sugars, and sodium’ [116]. Industrialized food or ultraprocessed food will be used interchangeably), may result in an overload of nutrients correlated with development of obesity and a lack of essential nutrients and micronutrients known to act against obesity [11]. Without discriminating the sources of calories, reducing the calorie intake usually results in a short phase of rapid weight loss, although the loss is not necessarily one of accumulated fat, but rather of fat-free mass [12]. Given that the main problem in obesity is, however, accumulated fat, losing any mass other than fat may be unproductive and not desirable.

Assuming that to respect a negative calorie balance could indeed be useful for individuals to lose weight, food information on energy intake and tools to measure the energy expense should be available, and they are. However, in reality it appears quite difficult for an individual to achieve this balance, because a constant error of only 175 kcal per day would have a considerable impact of up to 3 kg lost or gained within a year [13,14]. The available ways to measure caloric intake and expenditure cannot overcome this margin of error, as will be discussed in the following sub-sections.

### Energy intake and front label information

Front labels on food are meant to inform consumers’ choices, including those who may wish to count their calorie intake. However, some industrialized foods have shown differences between the content of energy claimed on their front label and analyses by a third-party, e.g. a health organization [15,16]. Similarly, the caloric content of the dishes in a restaurant menu may be underestimated by either the individuals or the supplier [17,18]. However, it seems more problematic that this information may not be understood correctly, if individuals do not have a certain minimal knowledge of nutrition [19,20]. In addition, when one self-assesses energy intake, under- and over-reporting consumption may occur [21]. Several technological devices have been developed to assist assessment of energy intake to reduce these biases, e.g. chewing count devices, wrist moving devices, or special digital photographic cameras for food. However, these devices may not yield sufficiently valid data [22–25]. Therefore, measurements of the energy balance may not meet the aforementioned narrow margin of error of 175 kcal per day.

### Physical activity and energy expenditure within concept 1

There are also barriers to assessing physical activity. The first one is the lack of standardized definitions of its levels, i.e. moderate, intermediate or vigorous intensity. Every level implies a different energy expenditure that also depends on other factors such as duration, body size and age. Hence, it is difficult to agree on which kind of physical activity should be recommended for effective weight control [26]. Whereas some studies and guidelines recommend vigorous physical activity [27,28], other evidence suggests that low-intensity activities such as walking, yoga, meditation and stretching are equally effective in reducing weight [28–30]. Second, energy expenditure might be assessed through technological tools aiming to enhance accuracy, such as pedometers and accelerometers, which measure walked distances and estimate the energy expenditure based on the weight, age and height of an individual. These measurements, however, have already an error of about 100 kcal/day [13]. Finally, despite the common belief that physical activity could equilibrate an energy imbalance, this is in reality difficult to achieve. For example, adults who eat a sandwich containing around 290 kcal should have to walk for around 90 minutes or almost 5 kilometers [31].

The energy expended in non-exercise activities that are part of neither the basal expenditure nor the digestive process is known as ‘non-exercise activity thermogenesis’ or NEAT [32] and also plays a role in the energy balance. These activities include ‘energy expenditure of occupation, leisure, sitting, standing, walking, talking, toe-tapping, playing guitar, dancing, and shopping’ [32], among others. The multitude of elements involved in NEAT and their variability make it even more challenging to assess them than physical activity, and hence, for them to be considered in the energy balance.

Despite the consensus of the health benefits of physical activity [33–35], independently from its suggested reduction of fat mass [36], there is controversy about whether sedentary behavior is directly involved in obesity development or not [37]. The relation between obesity and lack of physical activity seems not to be strong [38,39], and physical activity appears not necessarily to be a useful tool to ‘increase energy expenditure’ [40–45]. Nevertheless sedentary behaviors and reduction of physical work in jobs have been consistently stated as major factors for the development of obesity in modern societies [46].

## Possible consequences of using concept 1

### Ethical considerations on obesity and overweight

‘Eat less, move more’ appears as the most feasible solution to overweight and obesity and both possibilities seem to be within people’s reach [47]. The solution gives the impression of being so simple and straightforward that presumably all that are needed are willingness and self-control. Failing to revert obesity or overweight may be interpreted as lack of character, as one study has shown [48]. Concept 1, thus, may lead to blame and even stigmatize people with these conditions, e.g. punishments such as imposing a special tax on people with overweight and obesity using airplanes are already being discussed in both academia and mass media [49,50]. However, it may be ethically doubtful to attribute full responsibility to people with obesity and overweight, when individuals do not have full control over their food availability or accessibility [51,52]. In addition, it is thought that people may become obese through different pathways that possibly are independent of caloric balance, e.g. brain mediation of body fat mass, diminution of muscle mass and strength, as well as gut microbiota [53–55]. Therefore, it would be more ethical to use a ‘cause of obesity’ concept that does not transfer total responsibility to the individual.

### Public health strategies based on concept 1

Most anti-obesity programs are based on concept 1 or focus at least on one of its elements [56,57]. Possibly as a result, within certain populations, the bigger the average BMI the higher is the prevalence of dieters or exercisers [58]. However, the obesity epidemic continues to grow worldwide, and under the current trends, the chance to reverse it is virtually zero [59]. If apparently people and governments do what they are supposed to do to tackle the epidemic, but only isolated and not sustained results are achieved [60,61], it may be possible that the symptoms are being treated instead of the roots [62].

The use of concept 1 may have certain advantages, e.g. it is easy to understand, it has a straightforward logic, it creates awareness on food consumption and it can reinforce discipline. However, the disadvantages of the concept may outperform its advantages under the current global circumstances. Continuing to base strategies solely on concept 1 may, therefore, not only be ineffective in terms of public health, but also be an inefficient use of resources.

### Food industry and obesity

The participation of the food industry to reverse the obesity epidemic would be crucial [63,64], because it plays a major role in people’s choices and perceptions of food through its products and its marketing [65]. For example, the food industry communicates characteristics of its products through front label information, influencing consumption decisions [65]. However, most of the companies that dominate the global food market seem to focus only on meeting specific growth targets [66], overlooking the impact of their strategies on people’s health and weight, because the calories can be balanced anyway.

Subsidies from government to agricultural production are commonly based on the economic incentive of the volume of pruchase by the food industry, turning out certain raw materials such as sugar cane, cornand palm oil, cheap for the farmers to produce and sell to the food industry. Although such subsidized agricultural production is not a problem by itself, these materials are turned into harmful ingredients once they are ultra-processed by the industry [67], e.g. refined sugar, corn syrup and hydrogenated fats, all of which have proven to be closely related to obesity and to development of obesity-related chronic diseases [67–70]. Under concept 1, the effect of these foods on people’s health may be overlooked because individuals would self-regulate only their calorie balance.

Thus, the food industry has a share of responsibility within the obesity epidemic that may not be evident, because concept 1 transfers the responsibility of regulation to the individuals. By placing prime importance on the calorie balance, therefore, the food industry would always have a way out for not participating actively in a common frontline to tackle the obesity epidemic, which our concept 2 may avoid. The next section will propose an approach which differs from the calorie concept and might lead to more efficient actions aiming to tackle overweight and obesity.

## An alternative approach to causes for obesity

Different classes of food influence differently the energy balance. For example, if an individual follows a 2000 kcal diet with 55% of the total kcal from CHO, 30% from fat and 15% of protein, and if thermogenesis is taken into account, the remaining energy would be 1825 kcal and not 2000 kcal. That is because the body would use approximately 175 kcal out of those 2000 kcal only for catabolizing the macronutrients [4]. The energy needed for catabolism differs for every kind of macronutrient during the digestion process [9].

Therefore, it may be also possible that diet composition affects the whole digestive process and, hence, fat production and accumulation [53,71]. Given that obesity is an ‘abnormal or excessive fat accumulation that may impair health’ [1], it would appear appropriate to focus on the details of fat production and accumulation. This view may strengthen the strategies aiming to reverse the obesity epidemic.

### Lipogenesis

The production of fat is called lipogenesis. The body uses fat in different ways, but mostly as fuel [72]. Lipogenesis is commonly balanced with fat usage. Obesity is not a rapidly developing condition, but develops only if more fat is being produced than used – hence stored – continuously [73].

Potentially, all the macronutrients could be used by the body to produce fat. However, proteins are normally converted into amino-acids or peptides, and only exceptionally into fat [74,75]. Protein intake may also play an indirect role in lipogenesis through different hormonally controlled digestive processes [76]. Thus, it is normally considered that lipid production and storage mostly originate either from direct intake of fat, or from a process known as de novo lipogenesis, which is the transformation of CHO into fat [77].

### Fat metabolism

Fat is normally ingested in the form of triglycerides (TG). Fat needs to be oxidized into free fatty acids (FFA) to be used as energy [78,79]. This can happen through two different pathways, either directly from fat in food or from fat in adipose tissue. Insulin plays a key role to determine which pathway dominates [80]. That means that when insulin is high due to food intake, the breakdown of fat from adipose tissue stops and fat storage starts [73,81]. Conversely, when the insulin level is low, e.g. during a fasting state, the body makes use of the adipose tissue to generate energy [79]. Thus, energy production is regulated between fat storage in the presence of food and fat degradation when fasting.

### CHO metabolism

Digestible CHO are degraded to their simplest form, i.e. monosaccharides, mainly glucose. That is why such CHO are also called glycemic. Non-digestible CHO, namely fibers, are non-glycemic. Therefore, CHO can be classified according to whether or not they lead to raised glucose levels in the blood. How fast a glycemic CHO leads to a raised glucose concentration in blood is measured by its glycemic index (GI). CHO that are rapidly degraded and hence quickly raise the blood sugar level have a high GI, whereas the slowly degraded ones have a low GI.

Whenever a macronutrient is consumed, there may be an insulin response. However, the highest and longest secretion of insulin is stimulated by CHO with a high GI. Such CHO are common constituents of ultra-processed products. Insulin is a key to gear the metabolic processing of high GI carbohydrates into glucose (for immediate energy) or to build up fat reserves (for future energy needs). The latter pathway may be favored by some monosaccharides, e.g. fructose [82]. In both cases, a high and prolonged release of insulin normally takes place which relates to fat production and accumulation [83]. Whether CHO with high GI are consumed as liquid, e.g. sugary drinks, or solids, e.g. sugary cakes, seems to provoke a different insulin response, hence, different lipogenesis reactions. The liquid forms of CHO appear to be the most obesogenic form of high-GI carbohydrates [84,85]. The responses of insulin to food intake may be also influenced by genetic characteristics [86], suggesting that certain populations could be more vulnerable to overweight and obesity depending on their food availability [86].

### Negative cycle: insulin-overweight

Insulin plays a role in the process of lipogenesis and subsequent fat storage. This is also a self-regulated system of energy utilization. A problem may occur, however, if the system loses its self-regulation capacity, e.g. by a constant stimulation and consequent high presence of insulin due to the diet composition, and not necessarily by its overall caloric content. This disturbance of self-regulation may result in ‘more fat in and less fat out’. Additionally, a constantly high presence of insulin is known to provoke insulin and leptin resistance. Insulin resistance turns into a higher risk of developing diabetes type II, as several studies have shown [74,87–93], whereas leptin resistance may impede the body from using the stored fat as a source of energy [94].

We hypothesize that food composition could push an individual into a negative cycle: Under a constantly high presence of insulin, the body may increase its capacity for fat production and storage. As a result, the body grows bigger, and this leads to higher energy demands. If the body constantly stores fat but does not use it as a source for energy, it would not only grow bigger but also become constantly hungry. This would possibly cause a repeated intake of the diet that is at the origin of the imbalance, thus making the body even bigger and hungrier and so on. In this situation, ‘laziness’ and ‘gluttony’, i.e. concept 1, would actually be the symptoms of obesity and overweight, whereas the cause would include a hormonal imbalance, i.e. concept 2. This represents a situation that can be aggravated for populations with genetic predisposition to lipogenesis and fat storage, i.e. high insulin responses [86].

**Figure 1.**
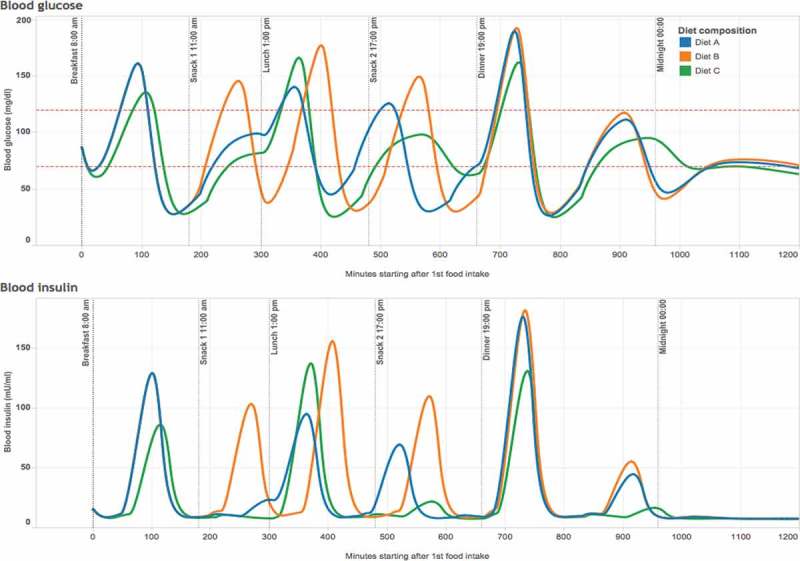
Differences in levels of blood glucose (top) and blood insulin (bottom) in the human body as provoked by diets with identical caloric content, but different compositions of macronutrients. Notes: Diets were assumed in this model calculation as follows, **Diet A (blue)**: 1670 kcal per day of energy content (7000 kjoules) with a composition of 53% CHO, i.e. 218 grams of CHO; 22% protein; and 25% fat. The CHO content is divided among three equal intakes during the day, i.e. 72.6 g for breakfast, 72.6 g for lunch and 72.6 g for dinner. **Diet B (orange)**: same composition as diet A but adding two snacks, i.e. one apple between breakfast and lunch (34 g of CHO) and one banana between lunch and dinner (30 g of CHO). **Diet C (green)**: the same energy content as in other diets, 1670 kcal per day (7000 kjoules), but with a composition of 38% CHO, i.e. 156 grams of CHO; 32% protein; and 30% fat. The CHO content is again divided into three equal intakes during the day, i.e. 52 g in breakfast, 52 g in lunch and 52 g in dinner. All diets were modeled for a non-diabetic adult of 60 kg of body weight using the web-based simulator of glucose and insulin levels in the human body ‘Glucosim’ developed by the Illinois Institute of Technology [95].

An insight into the insulin reaction provoked by different diet compositions with the same caloric content is shown in Figure 1. The graph was generated using data from the simulator of glucose and insulin levels in the human body, developed by the Illinois Institute of Technology [95]. This simulator works based on a mathematical model considering the physiological, metabolic and chemical reactions of the human body upon carbohydrate intake [96]. The depicted differences in insulin levels provoked by different diet compositions are apparently considerable.

It is worth noting the insulin reaction provoked by fruit consumption (Figure 1). This reaction may be considerably overestimated by the simulator because it does not differentiate between types of CHO. However, fructose as found in fruits may not be comparable in terms of either quality or quantity in reality to that found in industrial products [97]. There is indeed an actual controversy about the role of fruits in overweight and obesity, due to their fructose content and its role in fat production [98]. While some researchers have provided evidence on successful weight loss diets when removing fruits, fruit juices and other sources of fructose [99], others have argued that whole fruits do not cause overweight because their flavonoid and polyphenol content regulates sugar absorption [100]. Nonetheless, regarding the controversy, there is an apparent consensus in that the industrialized form of fructose (commonly sucrose mixed with high-fructose corn syrup) is the one mostly linked to obesity [82,97,101,102].

Macro-level studies may also support the latter assumption. For instance Riera-Chrichton and colleagues [103] analyzed population data from the USA between 1974 and 2006 and from 164 countries between 2001 and 2010. They determined that the strongest association between a specific type of macronutrients and development of obesity was with CHO, although they did not clearly distinguish what kind of CHO and, hence, the association is too broad given the different types of CHO. Similarly, Austin and colleagues [93] analyzed population data from the U.S.A between 1971 and 2006 and found that the increase in obesity prevalence was closely related to a higher consumption of CHO, again without any distinction of the types of CHO. Other studies and meta-analyses have found similar results [61,104,105]. Processed food apparently induces continuous insulin production in the human body, possibly due not only to large amounts of highly glycemic ingredients but also to the resulting diet composition, in terms of macronutrients, from consuming these products [76,83,106–108].

In summary, macronutrients influence fat production and accumulation either by a direct response provoked solely by intake, e.g. of CHO, or by hormonal regulation of the digestive processes, e.g. of protein and fat. A detrimental diet composition may produce a hormonal imbalance that could turn into a negative loop where individuals accumulate fat, but are not properly nourished which makes them constantly hungry, causing them to eat more from that diet composition. The resulting trap is reinforced by the modern food availability. Diet composition, thus, possibly has a larger influence on obesity and overweight than the calorie balance.

## A proposed revision of causes for obesity

We propose to revise the current concept of causes for obesity. Instead of placing prime importance on the energy balance, i.e. concept 1, we suggest to emphasize the effect of food on the metabolism. The guiding principle should be: ‘Overweight and obesity are determined by a hormonal imbalance characterized by a constant and sustained secretion of insulin, which results from particular dietary compositions. These are closely linked to modern food habits.’ In contrast to concept 1, this revision accounts better for recent insights into biochemical and hormonal pathways, is consistent with epidemiological and behavioral evidence, and may allow for more adequate concepts and actions in public health to reverse the obesity epidemic.

In regards of public health strategies, the revised concept 2 would associate the problem predominantly with an imbalance of hormonal processes that are biologically common to all humans, and which normally control a balanced insulin action on fat metabolism. As a result, leading public organisms may focus on tasks and solutions to reduce food-induced insulin overproduction that could be adopted and applied in contexts of their respective influence [109]. Targeting actions toward insulin-related metabolic processes could also lead to more homogeneous evidence to tackle obesity and overweight. This may also increase efficiency within the actions to reverse the obesity epidemic by building on a reliable ground [110]. Because the costs of obesity, including actions against it, are estimated at 2 trillion USD per year on the global gross domestic product (GDP) [111], even a small improvement in efficiency of actions could have a huge impact on savings.

Regarding ethics and stigma, the understanding of concept 2 may distribute responsibilities among the stakeholders, as opposed to concept 1 where corrective and preventive actions are attributed solely to the individual’s will. However, the individual dietary composition depends greatly on the availability and accessibility of food. These, in turn, are the result of regulations, economic incentives and food security, among other factors [112] that are out of individuals’ reach. For example, under concept 1, the minimal responsibility of government would be to ensure food availability and accessibility regardless of its composition, and provide knowledge about concept 1. Hence, the individual would have the option to self-achieve her or his calorie balance. Under the same scenario, the minimal responsibility of the food industry may be to support availability, accessibility and calorie balance knowledge. By contrast, under concept 2, a government’s minimal responsibility would be to ensure the availability and accessibility of such food that does not create havoc in the hormonal regulation of digestive processes along with knowledge of how a diet’s composition may affect individuals’ health. The food industry’s minimal responsibility would be to support the availability and accessibility of that non-harmful food, as well as supporting the knowledge about diet composition. Under this scenario, it would be difficult for the food industry and government to provide food that is marked as harmful by the knowledge that they also provide.

It is clear that consumers would always share in the responsibility about their food intake, but under concept 2, this responsibility may not remain exclusively with them. Clear areas of responsibilities may be drawn and stakeholders could be even made accountable for them. Hence, the revised concept may help to reduce, although not to eliminate, the negative connotations of an individual’s responsibility and the resulting stigmatization.

Guided by our proposed change of perspective, industry may start developing better products by paying attention to specific characteristics of industrial products, i.e. ingredients involved in hormonal imbalance. Moreover, the food industry may change its current defensive approach toward a more participative one. This could mean a significant and positive impact against the obesity epidemic. In addition, because all macronutrients have a role in insulin regulation, the proposed revision of the obesity concept may also have the advantage of not pointing to a single food and thus blaming a single item. This revision might even avoid a possible backfire in obesity prevalence caused by the control strategies, as may have happened when fat was stigmatized as the overweight promoter [88,113,114].

The proposed revision may have some disadvantages too. For example, people may find it harder to understand concept 2 rather than concept 1. Hence, educating people in concept 2 may be challenging. Another possible disadvantage is that because most of the current policies and supportive environment, e.g. front labels, mobile applications, textbooks, and Internet articles, are predominantly based on calories, concept 2 may lack initial support. Thus, an individual following concept 2 could have the impression of following a mistaken concept. However, it is possible that the advantages of adopting concept 2 may outperform its disadvantages in the long run.

## Leading to change

In advocating for this revised view on causes of obesity, resistance within institutions and their networks is to be expected. Revisiting the causes of obesity may also be interpreted as a withdrawal of the original concept, because concept 1 is broader than concept 2. However, restating the obesity problem as a hormonal imbalance is not necessarily in conflict with concept 1. There is an overlapping area that can be used to integrate the proposed change of perspective with the prevailing concepts of causes for obesity. In fact, the foods most closely related to concept 2, i.e. simple CHO, are also commonly discouraged by concept 1, although for different reasons. Lucan and DiNicolantonio [55] analyzed certain areas of compatibility between what they call ‘calorie-focused thinking’ and ‘more-nuanced thinking’, namely concept 1 and concept 2. They found certain overlaps as illustrated in Table 1, particularly in encouraging vegetables and discouraging sugary drinks.

**Table 1.** Overlaps between calorie-focused thinking and more-nuanced thinking from Lucan and DiNicolantonio [55]. Reprinted with permission.

		Calorie-focused thinking	
		Encouraged	Discouraged
More-nuanced thinking	Encouraged	Most vegetables, legumes, whole fresh fruits and unprocessed or sprouted grains; lean meats, poultry, and fish; water and unsweetened tea and coffee	Nuts and nut butters; avocados, olives and olive oil; whole dairy; oily fish
	Discouraged	100% fruit juices; enriched breads and pastas; fortified breakfast cereals (e.g. corn flakes, crisp rice); low-fat dairy (including sugary flavored fat-free yoghurts)	Sodas and other sugar- sweetened beverages; candies; baked sweets; French fries and butter-fried foods, snack chips and other processed items

The encouragement to base diets on non-industrialized food, whole plants and certain lean meats, all of which do not overstimulate insulin production, is also compatible with concept 2. Most of the non-obesogenic diets, such as the Mediterranean diet, Paleolithic diet or low-glycemic diet, are, therefore, compatible with concept 2. Katz and Meller compiled an extensive review on this [115] and their findings are shown in Table 2. This could also mean that most of the foregoing obesity research framed as concept 1 would still be relevant after reframing the information into concept 2. Therefore, the resistance to change could be minimized.

**Table 2.** Compatibility between diets (adapted from [115]). Reprinted with permission.

	Low-carbohydrate diet	Low-fat/vegetarian/vegan diet	Low-glycemic	Mediterranean diet	Mixed/balanced diet	Paleolithic
**Emphasis on**	Restriction of refined starches and added sugars	Plant foods direct from nature; avoidance of harmful fats	Restriction of starches, added sugars; high fiber intake	Foods direct from nature; mostly plants; healthful oils, especially monounsaturated	Minimization of highly processed, energy-dense foods; emphasis on wholesome foods in moderate quantities	Minimal intake of processed foods. Maximization of natural plant foods and lean meats
**Compatible elements**	Limited refined starches, added sugars, processed foods; limited intake of certain fats; emphasis on whole plant foods, with or without lean meats, fish, poultry, seafood					
**Potential general consistency**	Food, not too much, mostly plants. Portion control depending on the quality of foods, because higher quality foods have the tendency to promote satiety with fewer calories. Although neither the low-carbohydrate nor Paleolithic diet need to be ‘mostly plants’, both can be					

## Conclusion

We have discussed that the use of calories as energy units and concept 1 may be relevant to some areas of public health, but this concept has apparently not provided an efficient framework against the obesity epidemic. It may even foster stigma, produce negative consequences to unrelated third parties and prevent tackling strategies from being efficient.

Given the big impact of obesity on global GDP, it is a necessity to look for strategies that may increase the cost-efficiency of actions. By revisiting the causes for obesity, especially those stated by the leading health organisms, this article makes the case to view obesity as a consequence of disturbing the auto-regulation of hormones related to fat production and accumulation by diet composition, in particular that produced by industrialized processed food. Among other advantages, this proposed concept may offer an opportunity for the food industry to play a more responsible role in controlling overweight. If we really want to succeed in tackling the obesity epidemic, we must stop treating its symptoms, and start treating its causes instead.

## Availability of data and material

The data analysis from Figure 1 is publicly available online at the ‘Tableau Public’ repository: https://public.tableau.com/views/Insulinandglucosemodels/Dashboard1?:embed=y:display_count=yes.

## Paper context

The calorie balance as the cause of obesity is more complex than an intake–expense relation. By revisiting general facts about fat generation and accumulation, we propose to update the perceived causes of obesity including diet composition and recent insights into biochemical and hormonal pathways. The disadvantages of the currently stated cause of obesity may outbalance its advantages. A revision of this cause may promote more efficient actions in public health to reverse the obesity epidemic.
